# Application of deep brain stimulation for the treatment of childhood-onset dystonia in patients with MEPAN syndrome

**DOI:** 10.3389/fneur.2023.1307595

**Published:** 2024-01-24

**Authors:** Jaya Nataraj, Jennifer A. MacLean, Jordan Davies, Joshua Kurtz, Amanda Salisbury, Mark A. Liker, Terence D. Sanger, Joffre Olaya

**Affiliations:** ^1^Samueli School of Engineering, University of California Irvine, Irvine, CA, United States; ^2^Research Institute, Children’s Hospital of Orange County, Orange, CA, United States; ^3^Department of Neurology, Children’s Hospital of Orange County, Orange, CA, United States; ^4^Division of Neurosurgery, Children’s Hospital of Orange County, Orange, CA, United States; ^5^Department of Neurological Surgery, School of Medicine, University of California Irvine, Irvine, CA, United States; ^6^School of Medicine, University of California Irvine, Irvine, CA, United States; ^7^Department of Neurological Surgery, Keck School of Medicine, University of Southern California, Los Angeles, CA, United States; ^8^Department of Pediatrics, School of Medicine, University of California Irvine, Irvine, CA, United States

**Keywords:** MEPAN, dystonia, deep brain stimulation, *MECR*, pediatrics

## Abstract

**Introduction:**

Mitochondrial Enoyl CoA Reductase Protein-Associated Neurodegeneration (MEPAN) syndrome is a rare inherited metabolic condition caused by *MECR* gene mutations. This gene encodes a protein essential for fatty acid synthesis, and defects cause progressively worsening childhood-onset dystonia, optic atrophy, and basal ganglia abnormalities. Deep brain stimulation (DBS) has shown mixed improvement in other childhood-onset dystonia conditions. To the best of our knowledge, DBS has not been investigated as a treatment for dystonia in patients with MEPAN syndrome.

**Methods:**

Two children with MEPAN were identified as possible DBS candidates due to severe generalized dystonia unresponsive to pharmacotherapy. Temporary depth electrodes were placed in six locations bilaterally and tested during a 6-day hospitalization to determine the best locations for permanent electrode placement. The Burke-Fahn-Marsden Dystonia Rating Scale (BFMDRS) and Barry-Albright Dystonia Scale (BADS) were used for preoperative and postoperative testing to quantitatively assess dystonia severity changes. Patient 1 had permanent electrodes placed at the globus pallidus internus (GPi) and pedunculopontine nucleus (PPN). Patient 2 had permanent electrodes placed at the GPi and ventralis intermedius nucleus of the thalamus (VIM).

**Results:**

Both patients successfully underwent DBS placement with no perioperative complications and significant improvement in their BFMDRS score. Patient 2 also demonstrated improvement in the BADS.

**Discussion:**

We demonstrated a novel application of DBS in MEPAN syndrome patients with childhood-onset dystonia. These patients showed clinically significant improvements in dystonia following DBS, indicating that DBS can be considered for dystonia in patients with rare metabolic disorders that currently have no other proven treatment options.

## 1 Introduction

Mitochondrial Enoyl CoA Reductase Protein-Associated Neurodegeneration (MEPAN) syndrome, also known as mitochondrial enoyl-CoA/ACP (acyl carrier protein) reductase (*MECR*)-related neurologic disorder, is an extremely rare pediatric metabolic condition caused by autosomal recessive mutations in the *MECR* gene (1). This gene mutation results in defective mitochondrial fatty acid synthesis and dysfunction of the respiratory chain and multiple mitochondrial enzyme complexes (1). MEPAN syndrome was entered into the Online Mendelian Inheritance in Man (OMIM) in 2016 as childhood-onset dystonia with optic atrophy and basal ganglia abnormalities (DYTOABG; OMIM 617282) (2). According to the MEPAN Foundation, by early 2023, more than 30 people had been diagnosed with MEPAN syndrome globally since its discovery in 2016 (3). This condition typically presents in children aged 1 to 6.5 years old (4) with clinical characteristics of dystonia, optic atrophy, and hyperintense T2-weighted abnormalities in one or several structures of the basal ganglia, including the caudate, putamen, and pallidum, although other neurological signs (e.g., epilepsy and dysarthria) may also be present (1, 4, 5).

Dystonia, characterized by involuntary muscle contractions resulting in abnormal movements and postures, is one of the most frequently occurring childhood movement disorders and is particularly challenging to treat, given its wide range of etiologies and phenotypes (6–8). In MEPAN Syndrome, dystonia may be a consequence of Purkinje cell death due to high gene expressivity of *MECR* in those cells or impaired ceramide and iron metabolism leading to impaired neuronal function (9). Pharmacologic therapies for dystonia are limited in their varying degrees of efficacy and tolerability (7). For patients who fail conservative treatment, surgery may be considered. Deep brain stimulation (DBS), a functional neurosurgical procedure effective for the treatment of essential tremor and Parkinson’s disease (10), has been adapted for a variety of movement disorders, including adult and pediatric dystonia (11–16). In previous studies, the primary target of DBS stimulation in dystonia was the globus pallidus internus (GPi) (11–16). GPi-DBS has shown high efficacy in treating some genetic pediatric-onset dystonias and lower efficacy in treating other secondary dystonias (11–13), demonstrating that exploration of alternative targets may be indicated in these secondary dystonias (17). Among the genetic causes of dystonia, the efficacy of GPi-DBS also varies based on the specific monogenic form, with generalized dystonia (DYT-*TOR1A*), myoclonus dystonia (DYT-*SCGE*), and X-linked dystonia Parkinsonism (DYT/PARK-*TAF1*) showing good outcomes and other isolated and combined dystonias (DYT-*THAP*, DYT-*GNAL*, DYT-*KMT2B*, DYT*-ATP1A3,* and DYT*-ANO3*) showing suboptimal responses (18). The efficacy of GPi-DBS has also been reported in dystonia secondary to other neurodegeneration with brain iron accumulation (NBIA) diseases such as pantothenate kinase-associated neurodegeneration (PKAN), showing a variable effect size dependent on typical versus atypical phenotypes of PKAN (19). Other target regions have shown efficacy in treating dystonia such as the thalamus (20–22) and subthalamic nucleus (STN) (23–25). Alternative DBS target regions, such as the STN, have also been beneficial in the treatment of PKAN and other mitochondrial disorders (26, 27). The discovery of non-pallidal DBS targets for movement disorders and the suboptimal response to pallidal DBS for some etiologies of dystonia has also motivated discussions of multiple-target DBS. For example, combinations of GPi and pedunculopontine nucleus (PPN) DBS have been used to treat gait disturbances in a late-stage Parkinson’s Disease patient, where single-target stimulation showed only mild improvement (28). Combinations of STN and PPN dual-target stimulation have also been reported in Parkinson’s disease (28), and combined thalamic and pallidal DBS has shown efficacy in complex ipsilateral dystonia (29), dystonic tremor (30), and clinically complex movement disorders with profound functional impairment (31). The use of deep brain stimulation in other neurometabolic conditions is largely unknown.

A previously reported DBS technique (32, 33), based on the use of stereoelectroencephalography (sEEG) depth electrodes that are routinely used to identify epileptic foci, was utilized to identify possible stimulation targets in both patients. Potential targets were identified by previous reports in the literature on efficacious DBS sites (34, 35) based on patient-specific symptoms, as outlined in previous descriptions of the staged DBS target identification method (32, 33, 36–39). Temporary depth electrodes were used for test stimulation and recording at multiple possible targets. Permanent electrodes were implanted based on clinically effective targets identified during testing with depth electrodes. To the best of our knowledge, there have been no publications reporting the use of DBS for the treatment of dystonia in MEPAN syndrome patients.

## 2 Methods and materials

### 2.1 Patient selection

Two patients were retrospectively identified for this study based on their genetic composition and clinical features. The patients were siblings who were previously diagnosed based on whole-genome sequencing, which revealed two compound heterozygous variations in *MECR* (GenBank: NM_016011.3) in both patients: a pathogenic c.830 + 2dupT intron splice site variant that has been previously reported (1) and a variant of uncertain significance c.-39G > C. The initial presenting symptom in both patients was stiffness at the age of 7–8 months with continued worsening of symptoms throughout childhood, with patient 1 demonstrating more rapid progression following a mild viral exanthem at the age of 18 months. Both patients were engaged in therapies and the utilization of adaptive devices since infancy but had continued slowly progressive dystonic posturing as well as increasing fatigue, which was particularly prominent in patient 2. However, sleep was unaffected in both patients. Magnetic resonance imaging (MRI) in both patients revealed T2 hyperintensity in the bilateral caudate and putamen. The lab workup before diagnosis included whole-exome sequencing, thyroid studies, plasma amino acids, urine organic acids, acylcarnitine, serum lactate, hepatic function, and renal function. Lab studies were unrevealing with non-specific findings of mildly elevated pristanic acid, phytanic acid, 3-methyl glutaconic acid, and pyroglutamic acid. Cardiac and pulmonary workups were normal. No dysmorphic features were noted. Both patients were noted to have significant dystonia though with different clinical features. Patient 1 had axial and appendicular posturing, while patient 2 had appendicular posturing and greater hyperkinetic dystonic overflow. Patients were noted to have increased difficulty with ambulation with assistance (patient 1), self-feeding utilizing adaptive devices (both patients), and the utilization of adaptive communication devices (both patients) over the past 24 months before presentation for surgical consideration. These patients had previously undergone conservative measures such as pharmacotherapy, including benzodiazepines, carbidopa-levodopa, ropinirole, baclofen, tetrabenazine, trihexyphenidyl, and a ketogenic diet without benefit. Botulinum toxin injections were also unsuccessful in reducing dystonia. Dietary supplementation with triheptanoin was discontinued due to a lack of appreciable improvement. The patients were maintained on alpha lipoic acid and medium-chain triglyceride oil due to possible neuroprotective benefits but with continued significant dystonia. The patients’ parents requested the consideration of a neurosurgical procedure for the treatment of their dystonia given the continued progression. The parents provided consent and patients assented through standard hospital processes for DBS as a surgical treatment option for refractory dystonia. Patients 1 and 2 were 10 and 9 years old at the time of DBS surgery, respectively. Research consent was obtained for data collection only. Parents and patients provided consent for this publication.

### 2.2 Surgical procedure

#### 2.2.1 Phase 1 surgery (temporary electrode placement)

Stereotactic placement of temporary depth electrodes was performed to optimize the permanent implant location given unknown targets and the response of stimulation in MEPAN patients. For each patient, a preoperative stereotactic brain MRI with and without contrast with high-resolution T1 and T2 sequences was obtained. Stereotactic guidance for electrode placement was performed using a ROSA surgical robot with guidance from ONE™ software (Zimmer Biomet, Montpellier, France). Twelve Adtech MM16C depth electrodes (Adtech Medical Instrument Corp., Oak Creek, WI, United States) were placed at the identified targets. Target locations for temporary electrode placement included the bilateral ventralis oralis anterior/posterior (Voa/Vop), ventralis intermedius (VIM), and ventralis anterior (VA) nuclei of the thalamus as well as three targets in the GPi numbered anterior to posterior. Targeting used the standard surgical anatomical Schaltenbrand-Wahren atlas locations based on AC-PC coordinates with adjustment based on the patient’s anatomy. AC-PC coordinates for temporary electrodes are shown in Table 1. Each electrode was 1.2-mm in diameter and included 6 low-impedance (1–2 kOhm) contacts that were situated circumferentially around the electrode in a 2-mm band for stimulation, separated by a 5-mm distance between the centers. Ten high-impedance (70–90 kOhm) recording contacts, approximately 50 μm in diameter, were arranged in groups of two or three circumferentially around the electrode near the tip and between the low-impedance electrode bands. The distal contacts on the Voa/Vop lead were placed to extend into the STN, and the distal contacts of the VIM leads were placed to extend into the PPN, which was identified as a potential stimulation target for the treatment of axial motor symptoms because of its involvement in the production of movement and gait and positive outcomes reported in Parkinson’s disease (40). Electrodes were fixed to the skull using 13-mm anchor bolts (Adtech LSBK1-BX-06). Cefazolin was administered perioperatively to prevent infection. A postoperative CT scan was performed to ensure targeting accuracy and to rule out implantation-related hemorrhage or other complications.

**Table 1:** AC-PC coordinates of depth and permanent electrodes.

	Patient 1 depth electrodes		Patient 2 depth electrodes		Patient 1 permanent DBS electrodes		Patient 2 permanent DBS electrodes	
	Right	Left	Right	Left	Right	Left	Right	Left
GPi1 (anterior)	x = 16.11	x = −15.59	x = 16.22	x = −16.5		x = −15.85	x = 15.34	x = −17.85
	y = 5.14	y = 5.93	y = 4.25	y = 3.51		y = 7.95	y = 7.45	y = 5.79
	z = −5.19	z = −5.75	z = −5.97	z = −6.59		z = −4.01	z = −4.45	z = −2.77
GPi2	x = 19.35	x = −18.02	x = 19.49	x = −18.17				
	y = 1.82	y = 5.34	y = 2.37	y = 2.75				
	z = −5.95	z = −5.41	z = −5.6	z = −5.47				
GPi3 (posterior)	x = 21.41	x = −23.49	x = 21.76	x = −21.48	x = 21.45			
	y = 0.90	y = 2.56	y = −1.24	y = 0.62	y = 5.19			
	z = −5.53	z = −5.69	z = −4.74	z = −6.32	z = −4.90			
VA	x = 4.57	x = −6.13	x = 4.67	x = −5.77				
	y = 0.38	y = −1.07	y = −1.32	y = −4.65				
	z = 1.98	z = −0.11	z = 1.50	z = 0.95				
VIM-PPN	x = 6.00	x = −4.68	x = 5.09	x = −4.78				
	y = −15.22	y = −14.28	y = −14.81	y = −16.89				
	z = −13.91	z = −16.10	z = −15.89	z = −15.23				
PPN^a^					x = 4.04	x = −3.29		
					y = −17.21	y = −16.78		
					z = −13.04	z = −16.74		
VIM^a^							x = 8.68	x = −7.02
							y = −7.68	y = −10.64
							z = −5.00	z = −5.63
Vo-STN	x = 11.5	x = −11.72	x = 11.47	x = −10.60				
	y = −3.69	y = −2.78	y = −4.22	y = −4.73				
	z = −3.74	z = −5.29	z = −4.82	z = −5.23				

a Only contact spacing on the sEEG electrode allows concurrent stimulation targeting of the VIM and PPN. Due to permanent electrode contact spacing, a single target (VIM or PPN) had to be selected.

#### 2.2.2 Neuromodulation unit testing

After the depth electrodes had been inserted, the patients were moved to the “Neuromodulation Monitoring Unit (NMU)” in the Neurology Inpatient Unit for monitoring. Assessment began 24 h after the completion of surgery to allow recovery from general anesthesia.

After lead integrity had been confirmed by ensuring an impedance of less than 7 kOhm, bipolar stimulation was conducted through the adjacent low-impedance contacts on the MM16C electrodes. All stimulation and impedance tests were carried out using a Medtronic external neurostimulator 37,022 and Medtronic 8,840 DBS programmer (Medtronic Inc., Minneapolis, MN, United States). The stimulation was administered at bilateral (homologous left and right) contacts at a 90 μs pulse width and frequencies of 60 Hz and 185 Hz, and the voltage was increased up to 5 V in the pallidum, 3 V in the thalamus, and 1 V in PPN. Initial benefits, including reduced overflow, reduced tone, and an improvement in the range of motion in both patients, were identified after patients, their families, and an examiner utilized the finger-to-nose assessment. Subjective improvement in pain and ease of movement was also noted. Side effects were also recorded and, if occurring at or below the standard therapeutic voltages (lower than 3.5 V for pallidum, or lower than 2.5 V for thalamus), the area was excluded. It should be noted that the thalamic and brainstem stimulation effects could be seen almost immediately, while pallidal stimulation effects typically developed over several minutes.

Patient 1 was predominantly assessed during ambulation and fine motor tasks, including eating and utilizing a home communication booklet. Methylphenidate (10 mg) was utilized daily to promote wakefulness and hence allow an assessment of the test stimulation.

Patient 2 had no response to methylphenidate in promoting wakefulness, hence modafinil (100 mg) was utilized with an excellent response. Patient 2 was predominantly assessed in gross and fine motor tasks, including moving out of bed, utilizing an adaptive assistive communication device, and feeding.

It is important to note that the wakefulness-promoting agents methylphenidate and modafinil were used exclusively during the NMU hospitalization period to prevent unnecessary prolongation of hospitalization. To successfully identify clinically beneficial contacts, patients were asked to tolerate several hours of stimulation testing in the awake and alert states. Much of this testing involved physical activity such as reaching and ambulation. Stimulants were used to promote wakefulness to allow the completion of testing. The use of stimulants during functional testing is also often seen in stroke rehabilitation, where wakefulness-promoting agents are used in the stroke hospitalization period to improve discharge disposition, and in the post-hospitalization period to improve patients’ ability to participate in intensive rehabilitation (41). In both subjects, stimulants had been previously trialed before proceeding with deep brain stimulation for attention deficits and daytime fatigue, as identified by their primary care provider, with mixed benefits with respect to attention. Stimulant medication trials, including modafinil and lisdexamfetamine, have been trialed subsequently for these concerns postoperatively. Contacts were tested first bilaterally and then unilaterally, utilizing activities of daily living as previously identified above, with adjustments to the frequency by 5 Hz intervals and pulse width by 10 μs steps to find the optimal settings. As numerous contacts had to be tested at various frequencies, the process took 6 days for each patient. The effects of clinical simulation in thalamic, subthalamic, and brainstem targets were often seen within 3–5 s, as previously reported (42). While pallidal stimulation is noted to have longitudinal effects, immediate changes within 3–5 min can also be assessed, allowing for further programming of these beneficial contacts with multi-hour or overnight assessment. After the effective stimulation parameters had been identified, the same parameters were tested two times, at least 24 h apart, and confirmed by multiple members of the medical team to guarantee a consistent response. As both subjects had multiple beneficial targets identified (GPi, PPN, and VIM for patient 1 and VIM and GPi for patient 2), stimulation parameters were then trialed in multiple targets to assess if they provided additional benefits over a single target alone. Dual-target stimulation was noted to be more effective than single targets, both unilaterally and bilaterally in both subjects. Patient 1 had the best response to combined GPi-PPN stimulation regarding dystonic posturing, both axially and in extremities, versus combined PPN-VIM or combined GPi-VIM stimulation. Blinding to stimulation parameters was performed to remove bias by patients, parents, and team members. Stimulation was also evaluated overnight at both single and dual targets to investigate any long-term effects, including positive or adverse effects on sleep. No effects on sleep were appreciated in either patient.

Discrete activities of daily living, including feeding, ambulation, and communication, were assessed to determine which areas of stimulation provided the most functional benefit. While sufficient videos for scoring on the BADS and BFMDRS are obtained during the NMU testing, these scales are not performed routinely during this time, as, in our experience, the brief duration of stimulation in the NMU underestimates the long-term benefit seen with permanent DBS. Conversely, the absence of benefit with stimulation in the NMU indicates that long-term stimulation in these areas will not provide significant benefit, eliminating them from consideration for permanent implantation. Combined GPi-PPN stimulation benefited the axial posturing and hypertonic dystonia in patient 1, whereas combined GPi-VIM stimulation benefited the hyperkinetic overflow and appendicular posturing of patient 2 the most.

Following the completion of testing, the electrodes were removed under general anesthesia. Both patients were observed overnight to ensure adequate recovery and were discharged the following morning. Patient 2 was shown to have partially devitalized tissue at the area of the initial lead implant; hence, phase 2 surgery was held for an additional 4 weeks to ensure complete healing without any evidence of infection.

#### 2.2.3 Phase 2 surgery (permanent DBS electrode placement)

**Figure 1:**
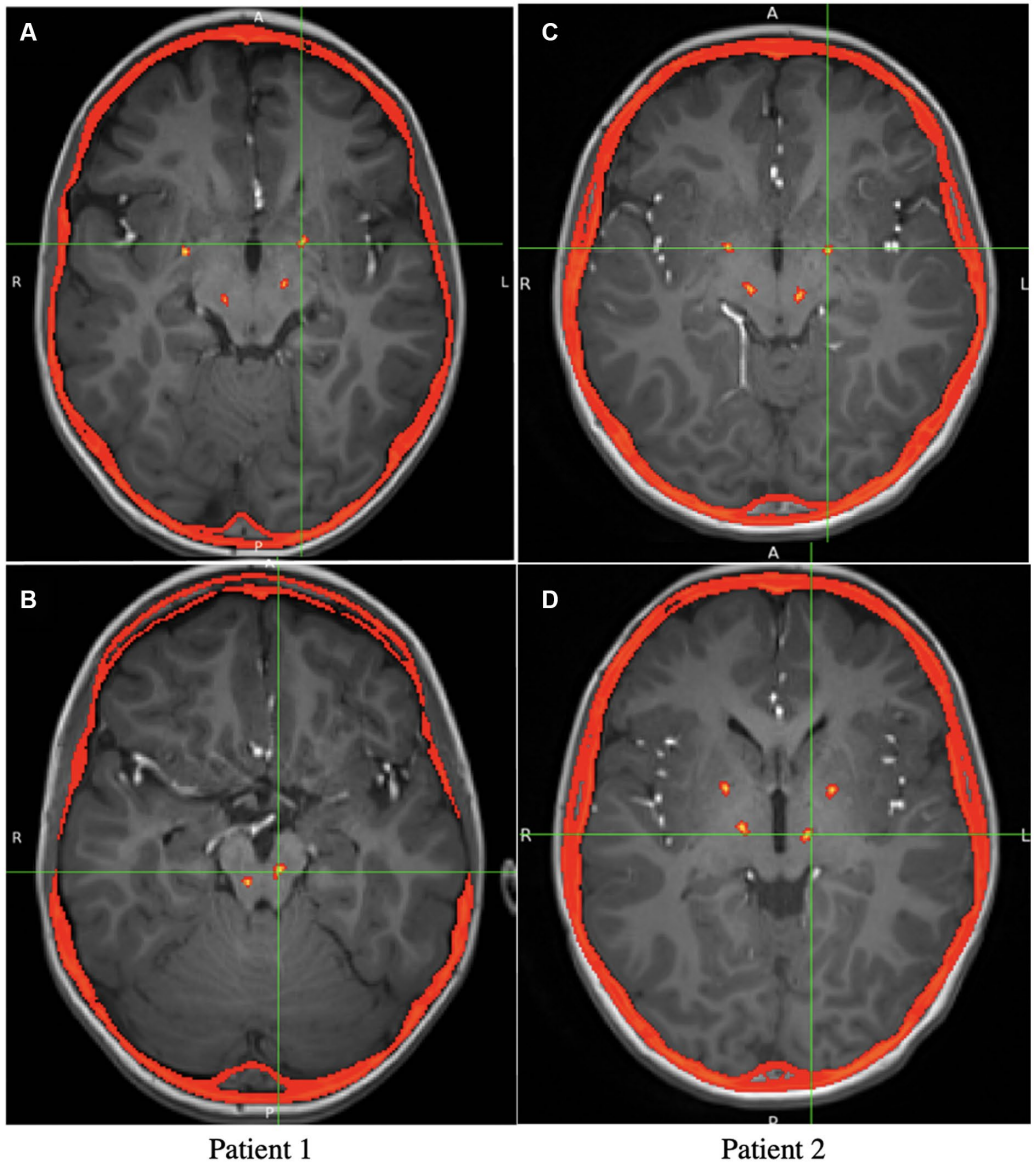
Axial view of the postoperative CT overlaid on preoperative MRI, showing the locations of the Medtronic leads. **(A)** Axial view of the GPi electrodes in patient 1 (The PPN electrodes are also visible but not at the target). **(B)** Axial view of the PPN electrodes in patient 1. **(C)** Axial view of the GPi electrodes in patient 2 (The VIM electrodes are also visible). **(D)** Axial view of the VIM electrodes in patient 2 (The GPi electrodes are also visible).

At least 14 days following depth electrode removal, permanent lead implantation took place under general anesthesia using procedures similar to those used in the initial surgery. Targeting was determined by merging the preoperative MRI and postoperative first-phase CT scan with the second-phase CT performed with bone fiducials in place. To place two electrodes in each hemisphere, the entry point on each side was adjusted so that both entry points on the same side could fit through the same burr-hole and be held in place with the same Sensight Burr Hole Device (Medtronic Inc.). For both patients, four Medtronic Sensight 1.5-mm electrodes were utilized. The extracranial leads were “booted” as per the manufacturer’s instructions and placed under the skin before the wound was closed. A postoperative CT scan was performed to rule out implantation-related hemorrhage or other complications and subsequently merged with the preoperative MRI to ensure accuracy in targeting, as shown in Figure 1. Perioperatively and for 72 h postoperatively, intravenous vancomycin and ceftazidime were administered for infection prophylaxis. After finishing the intravenous antibiotics, the patients were discharged with 10 days of enteral dicloxacillin. The AC-PC coordinates for permanent DBS electrodes are shown in Table 1.

#### 2.2.4 Phase 3 surgery (pulse generator placement)

Approximately 2 weeks after the second-phase surgery, Medtronic B34000 Sensight extensions were connected to the intracranial electrodes and tunneled subcutaneously to implanted pulse generators (Medtronic Activa RC) placed in the chest. To ensure programming with similar frequencies in homologous targets, homologous leads were routed to the same stimulator (e.g., both VIM/PPN leads to the stimulator in the right chest, and both GPi leads to the stimulator in the left chest). Perioperatively, intravenous cefazolin was administered. Both patients were discharged on the same day, with 10 days of oral dicloxacillin prescribed post-discharge.

### 2.3 Outcome measures

The severity of each patient’s dystonia was assessed utilizing the Burke-Fahn-Marsden Dystonia Rating Scale Motor Component (BFMDRS-M) (43, 44) and the Barry-Albright Dystonia Scale (BADS) (45) on video recordings preoperatively and approximately 12 months postoperatively, with timings based on the patient’s scheduled follow-up visits in clinic. Both scores were performed by a single clinical staff member and confirmed by video independently by a pediatric movement disorder physician. Both scorers agreed on all scoring. The videos could not be blinded as both patients showed visible aging and growth.

## 3 Results

Both patients were brought in for mapping of their implanted electrodes at 3–4 weeks following neurogenerator placement. They returned to the clinic every 2–4 weeks for further programming for the first 3 months and then every 3–6 months, depending on the response. Patient 1 required more frequent programming visits over the first year due to adaptation to the programming settings in the PPN after several weeks. During this time, no significant pharmacotherapy changes were made to limit confounding effects. Patient 1 did not tolerate typical therapeutic voltages due to worsening dystonic posturing with voltages above 0.5 in both the GPi and PPN, which is highly unusual but has been seen with other patients with stimulation in the PPN (38). Significant clinical effects regarding dystonic posturing were noted with changes of 0.05–0.1 V in both areas even at 1 year postoperatively, which were visible within minutes of changes to the PPN and hours to the GPi, suggesting that the patient remains highly sensitive to changes in these areas. Objectively, both patients demonstrated significant improvement in the BFMDRS, and patient 2 also demonstrated a mild improvement in the BADS. Given the severity of patient 1’s dystonia, his lack of change in the BADS was not unexpected as the overall improvement in his dystonia was noted in relation to decreased posturing with voluntary activation, which is more accurately captured on the BFMDRS-M. Patient 1 had a 34.9% decrease in the BFMDRS-M score. Patient 2 had a 49.6% decrease in the BFMDRS-M score and a 19% decrease in the BADS score. Scale scores and programming parameters at the time of scoring are shown in Table 2.

**Table 2:** Patients’ response to DBS.

	BFMDRS-M preoperative	BFMDRS-M postoperative	BADS preoperative	BADS postoperative
Patient 1	76.0	49.5^a^	23.0	23.0^a^
Patient 2	63.5	32.0^b^	21.0	17.0^b^

a GPi:1b-2a-2c- case + 0.2v/90usec/185 Hz // 8–9c-10c- case + 0.4v/90usec/250 Hz.

PPN (cycling with stimulation on for 1 min and off for 5 min): 1c- case + 0.2v/60usec/250 Hz // 9c- case + 0.2v/60usec/250 Hz.

b GPi: 0–1- case + 2.5v/60usec/140 Hz // 8–9-c + 2.7v/60usec/150 Hz.

VIM (cycling with stimulation on for 100 μs and off for 100 μs): 1–0+ 2v/60usec/90 Hz // 9–8+ 2v/60s/90Hz.

Subjectively, patient 1 appeared to show substantial improvement in axial posture with ambulation and while seated. His comfort when using his wheelchair and during travel was improved, as was his self-feeding. Patient 2 exhibited improved appendicular postures and improved overflow, allowing the improved ease of feeding, movement around the home, and use of his communication device. Parents self-reported improvement in patients’ and parents’ quality of life following the surgical procedure. These improvements are particularly notable given the degenerative nature of the patient’s underlying condition. No complications were noted for either patient.

## 4 Discussion

Treatment of childhood-onset dystonia secondary to MEPAN syndrome is exceptionally challenging due to the range of clinical characteristics seen in MEPAN patients and the neurodegenerative nature of the condition. Another layer of complexity is added by the variation in motor features associated with the movement disorder component of MEPAN, as observed in the two patients discussed in this study. MEPAN is an incurable progressive disorder, and the treatment of it and similar progressive diseases with DBS should be considered for long-term palliation, with the recognition that DBS neither slows nor reverses disease progression. These cases illustrate that, despite the progressive nature of the underlying disorder, significant short-term clinical benefits and improvement in the quality of life may make DBS worthwhile even when an ongoing injury cannot be stopped.

Although there are no current reports of neuromodulatory intervention in children with MEPAN syndrome, the efficacy of GPi-DBS has been reported for movement disorders in other degenerative neurometabolic conditions such as PKAN and mitochondrial disorders (19, 26, 46–48). GPi-DBS showed moderate efficacy in atypical PKAN, variable benefits in typical PKAN (19), and mixed effects in mitochondrial disease (26, 46–48). However, GPi-DBS may be less effective in cases of dystonia that present with structural abnormalities and will not affect the progression of the underlying metabolic condition. The tendency of MEPAN syndrome to present with basal ganglia abnormalities, in conjunction with the low prevalence of the disorder, indicates that standard DBS targeting procedures may not be suitable for those with MEPAN syndrome (4).

Alternative DBS targets such as the STN have been used to treat PKAN and mitochondrial disease as well and have shown some promise (26, 27). A previous case report suggests that DBS of targets identified by a staged procedure can provide benefits in movement disorders secondary to rare genetic or metabolic conditions where optimal targets are unknown (36). The response of the two patients in this study suggests that DBS can provide potential benefits for MEPAN syndrome, especially when options for standard pharmacotherapy treatment have been unsuccessful. The differing phenotypes of the disorder observed in each patient were associated with unique combinations of DBS targets, indicating that treatment efficacy is heavily reliant on target location. Furthermore, in this case of two brothers with the same genetic disorder, we observed that the optimal choice of target location is determined more by specific motor symptoms than by the underlying genetic etiology.

DBS mechanisms of action on dystonia are not well understood (49), but the GPi is thought of as the major output nucleus of the basal ganglia, modulating thalamocortical pathways via inhibitory projections to thalamic nuclei and the PPN. The improvement in Patient 1’s axial symptomatology with PPN-DBS could be attributed to its widespread connections to motor regions of the brainstem and spinal cord (50). Conversely, the identification of the VIM as optimal for Patient 2’s appendicular symptomatology is possibly attributable to the modulation of cerebellothalamic projections by DBS, such as those thought to participate in tremor genesis (30). The interplay between GPi-PPN and GPi-VIM stimulation also requires further understanding to ascertain if combined DBS may provide benefits across varying presentations of childhood-onset dystonia related to metabolic conditions and in which conditions each of these combinations may be optimal. DBS may also modulate neurotransmitter production and glucose uptake in regions local to the area of stimulation, which could drastically affect treatment outcomes in cases where an underlying metabolic condition exists (51–53). A greater understanding of the local and global effects of DBS is necessary in optimizing its use for the treatment of movement disorders related to complex genetic and metabolic conditions such as MEPAN syndrome (54).

This report is limited by the small patient population, the heterogeneity of dystonic features across subjects, and the lack of comparative literature regarding the utilization of DBS in MEPAN syndrome. Despite these limitations, it is important to note that both patients showed clinically significant improvements in dystonia scales 1 year postoperatively, without any appreciable complications. Testing suggested that likely targets may include VIM, PPN, and anterior and posterior GPi, depending on the phenotypic presentation of the condition (55). The formal quality of life scale obtainment preoperatively and postoperatively will also be essential in obtaining an objective analysis of response.

An important takeaway is the feasibility and importance of utilizing a staged procedure with sEEG testing in situations where targets for DBS are currently unknown. The difference in target efficacy in two brothers with the same mutation suggests that significant caution is needed when attempting to use a successful response in one patient to predict the target location in another. In this case, the clinical team had proposed using the outcome in one brother to guide implantation in the other and only tested both brothers independently (and within weeks of each other) at the parents’ insistence. While the implementation of a staged procedure requires immense resources in terms of the length of hospitalization, coordination of clinical care, testing, programming, and patient and parent cooperation, the identification of different optimal target locations in subjects with the same underlying etiology strongly demonstrates the need for this staged approach for patients with differences in disease presentation. The time burden and possible delay in treatment this paradigm places on patients and families should also not be underestimated. It is essential that both patients and parents are assessed for adequate social support or coping mechanisms before and throughout the procedure. Ethically, it is imperative that patients and families are made aware of alternatives and also the evidence supporting them, particularly in conditions where the targets for DBS are unknown. The response of these two patients indicates that the efficacy of this staged testing is not limited to a single clinical presentation and that a similar DBS procedure can be considered in other rare genetic and metabolic conditions for which there are no known treatments.

## Data availability statement

The data presented in this study are available on request from the corresponding author, subject to patient consent to privacy. The data are not publicly available due to patient privacy.

## Ethics statement

The studies involving humans were approved by Children’s Hospital of Orange County Human Subjects Institutional Review Board approval 200330, 13 July 2020 to 24 July 2024. The studies were conducted in accordance with the local legislation and institutional requirements. Written informed consent for participation in this study was provided by the participants’ legal guardians/next of kin. HIPAA authorization for use of protected health information was obtained.

## Author contributions

JN: Data curation, Formal analysis, Project administration, Writing – original draft, Visualization, Writing – review & editing. JM: Project administration, Writing – original draft, Conceptualization, Data curation, Formal analysis, Methodology, Writing – review & editing. JD: Writing – original draft, Conceptualization, Methodology. JK: Writing – original draft, Methodology. AS: Project administration, Writing – review & editing. ML: Conceptualization, Supervision, Writing – review & editing. TS: Conceptualization, Supervision, Validation, Writing – review & editing. JO: Data curation, Methodology, Supervision, Writing – original draft, Visualization, Writing – review & editing.
